# Glucocorticoids as Regulators of Macrophage-Mediated Tissue Homeostasis

**DOI:** 10.3389/fimmu.2021.669891

**Published:** 2021-05-17

**Authors:** David Diaz-Jimenez, Joseph P. Kolb, John A. Cidlowski

**Affiliations:** Molecular Endocrinology Group, Signal Transduction Laboratory, National Institute of Environmental Health Sciences, National Institutes of Health, Research Triangle Park, NC, United States

**Keywords:** glucocorticoids, macrophages, homeostasis, glucocorticoid receptor, inflammation

## Abstract

Our immune system has evolved as a complex network of cells and tissues tasked with maintaining host homeostasis. This is evident during the inflammatory responses elicited during a microbial infection or traumatic tissue damage. These responses seek to eliminate foreign material or restore tissue integrity. Even during periods without explicit disturbances, the immune system plays prominent roles in tissue homeostasis. Perhaps one of the most studied cells in this regard is the macrophage. Tissue-resident macrophages are a heterogenous group of sensory cells that respond to a variety of environmental cues and are essential for organ function. Endogenously produced glucocorticoid hormones connect external environmental stress signals with the function of many cell types, producing profound changes in immune cells, including macrophages. Here, we review the current literature which demonstrates specific effects of glucocorticoids in several organ systems. We propose that tissue-resident macrophages, through glucocorticoid signaling, may play an underappreciated role as regulators of organ homeostasis.

## Introduction

For over 70 years, synthetic glucocorticoids have been used to treat numerous inflammatory conditions, including allergies, asthma, autoimmune diseases, sepsis, and cancer. This is partially due to their profound ability to modulate the immune response through anti-inflammatory and immunosuppressive mechanisms. Endogenous glucocorticoids (such as cortisol in humans and corticosterone in mice) are a class of adrenal cortex steroid hormones regulated through the hypothalamic-pituitary-adrenal axis. They are produced in response to stresses such as infection, but are also naturally secreted in circadian and ultradian cycles. Cortisol acts as a biochemical signaling molecule and is involved in numerous metabolic processes in the body. However, cortisol deficiency in the body leads to an exacerbated inflammatory response. Furthermore it is well recognized that the serum level of cortisol in the body is decreased in the elderly.

Both endogenous and synthetic glucocorticoids (dexamethasone and prednisone, among others) exert their therapeutic effects primarily through the glucocorticoid receptor (encoded by NR3C1, hereafter GR), and their efficacy in controlling inflammatory conditions results from the pleiotropic effects of the GR signaling pathways (1). GR is a member of the nuclear receptor superfamily and is a ligand-dependent transcription factor. It is expressed ubiquitously in almost every human cell, including all immune cells (2). When bound by its ligands, GR translocates to the nucleus and occupies specific palindromic DNA sequences within the open chromatin, called glucocorticoid response elements (GREs), to activate or repress gene expression (3–8). GR activates gene expression through different mechanisms that involve direct binding of dimers to GREs within GR-binding sites or composite binding in which GR and another transcription factor interact with distinct response elements within the same genome location. For example, glucocorticoids enhance phosphoenolpyruvate carboxykinase gene expression through GR and CREB binding to GREs and a cyclic AMP response element, respectively, within close proximity (9).

Glucocorticoid-mediated gene repression or GR transrepression occurs through direct binding of GR to repressive DNA motifs (negative glucocorticoid response elements or nGREs) (10, 11) or tethered recruitment of ligand-bound GR to another transcription factor without DNA interaction. Tethering is likely the most studied mechanism for immune regulation by glucocorticoids. Many studies have linked this mechanism to the beneficial anti-inflammatory actions of glucocorticoids (12, 13). Tethering occurs when GR binds to another transcription factor without interacting with DNA. GR has been shown to tether key pro-inflammatory transcription factors, including nuclear factor-κB (NF-κB) and activator protein 1 (AP-1), which antagonizes their interaction with chromatin, influences the recruitment of co-regulators, and results in gene expression inhibition. Remarkably, using genome-wide profiling in LPS activated macrophages upon Dex treatment, Uhlenhaut et al. found that 20% of GR-dependent repression is related to nGREs and tethered sites, suggesting that the positive and negative GR cistromes are predominantly composed of classical GREs in close proximity to NF-kB and AP-1 binding sites (6).

Interestingly, GR*^dim^* mice carrying an amino acid substitution (A465T) in the D-loop of the DNA-binding domain of GR showed reduced, but not completely absent, transactivation ability in response to glucocorticoids (14), suggesting that the GR dimerization-dependent gene regulation was not essential for the effects of GCs. Direct binding of GR as monomers also has been described (5, 15). Initially, the mechanism of transrepression proposed that the monomeric state of GR repress the transcription by tethering to DNA-bound TFs (10, 11). Using mouse liver from WT and GR^dim^ under endogenous corticosterone exposure and chromatin immunoprecipitation with lambda exonuclease digestion and sequencing (ChIPexo), Lim HW et al., reported that monomeric GR interaction with a half-site motif is more prevalent than homodimer binding (5). This monomeric GR interaction with a half-site motif display greater cell-type specificity and enrichment for lineage-determining TFs relative to dimer sites. These data arguing in favor of a model termed half-site-facilitated tethering, where sequence-specific interaction of GR monomers to different motifs promotes transient contacts between monomers and nearby TFs (5).

The GR has been previously reported to modify chromatin structure as well (16–18). New evidence establish that glucocorticoids exert primary repressive effects on transcription through altering chromatin structure (18). For example, using a global run-on sequencing or GRO-seq, Sasse et al., demonstrated that the repression of many TNF-regulated genes and enhancers by dex treatment rapidly changes the chromatin structure in a process that does not required GR occupancy (18). This evidence suggest that either a transrepressive or nGRE mechanisms on the NF-kB signaling are not implicated. Moreover, the high resolution given by GRO-seq also allowed to discover a secondary anti-inflammatory effects resulting from transcriptional cooperation between GR and NF-kB at a subset of regulatory regions (18). This cooperative glucocorticoid-TNF crosstalk in the repression of inflammatory processes previously was observed by Vettorazzi et al. in a model of acute lung inflammation (7), where Dex and pro-inflammatory stimuli in macrophages, synergistically *via* GR increased sphingosine1-phosphate (SphK1) expression and the levels of S1P circulating that play a role in attenuating lung inflammation. These data provide evidence that reducing the expression of pro-inflammatory cytokines, a classic feature of glucocorticoids treatment, is not sufficient to resolve the inflammation.

Glucocorticoids do not only antagonize proinflammatory gene expression. They have recently been shown to induce proinflammatory gene expression in several cell types, including macrophages (19–21). For example, dexamethasone upregulated expression of the NLRP3 inflammasome in human THP-1 macrophages, causing them to be more responsive to the NLRP3 agonist ATP (19). In addition, co-regulation of genes by glucocorticoids and cytokines has been demonstrated in which glucocorticoids and cytokines synergize to enhance proinflammatory mediator production (20). Finally, GR-mediated induction of exopeptidase DPP4 contributed to the increased mobility of macrophages in response to dexamethasone (21). However, the extent of this co-regulation and its mechanism in immune cells is poorly understood.

Macrophages are innate immune system effector cells which, upon inflammation, phagocytose apoptotic and necrotic cells. They are involved in tissue repair and modulate inflammation by balancing pro- and anti-inflammatory responses. Interestingly, glucocorticoids seem to have limited efficacy in the control of inflammation in diseases related to macrophage activity, such as, atherosclerosis, ulcerative colitis and respiratory tract diseases (22, 23). While it is true that many of the diseases mentioned above are quite successfully controlled by corticosteroids treatment, this has been associated to early stages of the diseases because they are able to inhibit many components of the inflammatory response. Even in the clinical management of some of them, the use of corticoisteroids has been recommended as adjunct treatment at the lowest dose possible and for the shortest time possible. Although glucocorticoids induce cell death and reduce cell survival in immune cells such as T and B cells, macrophages are relatively resistant to glucocorticoid-induced apoptosis (2). These observations support the idea that the pro-inflammatory versus the anti-inflammatory regulatory actions of glucocorticoids may be predominant in macrophages.

The macrophage ontogeny has been challenged during the last two decades. The paradigm that tissue-resident macrophages are continuously replenished by blood-circulating monocytes, which arose from bone marrow (BM)-derived precursors was updated since Merad et al, showed that Langerhans cells, a kind of macrophages in the skin, were resistant to the irradiation and were not derived from donor after congenic BM transplanstation (24). The current models of macrophage ontogeny have been established through genetic fate-mapping techniques. For example, now is well-known that major tissue-resident macrophage populations, including microglia, liver Kupffer cells, lung alveolar macrophages, epidermal Langerhans cells and splenic macrophages, are established during the embryogenesis from the yolk sac (YS) anf fetal liver and subsequently maintain themselves independently of replenishment by blood monocytes during adulthood (25, 26). Contrary, macrophages population from the gut and heart are constantly replenished by blood monocytes postnatal (27, 28). In the new era of “omics” techniques, single-cell RNA-sequencing have revealed a next level of complexity to the functional heterogeneity of the embryonic origin of key tissue-resident macrophage populations. For example, depth analysis of arterial macrophages at single-cell resolution in steady state and in response to angiotensin-II (AngII)-induced arterial inflammation revealed dual origin of arterial macrophages from both YS and BM-hematopoiesis, a process that is stable in adult mice, but declines in numbers during ageing and is not replenished by bone marrow (BM)-derived macrophages (29). In AngII inflammation, BM-derived macrophages invade the inflamed adventitial tissue, while resident -YS erythromyeloid progenitors (EMP)-derived macrophages- were self-renewal and proliferate locally providing a distinct transcriptional profile linked to tissue regeneration (29). Despite the fact that our understanding of ontogeny of macrophages is increasing, the precise developmental trajectories of tissue-resident macrophages remain undetermined.

Another level of complexity into the macrophages biology is given by the activation or polarization processes. Macrophages are polarized according to changes in their environment and are classically divided in two main categories, M1 macrophages and M2 macrophages (30). M1 macrophages are mainly involved in pro-inflammatory responses, classically generated upon induction by microbial products, such as LPS and pectidoglycan and pro-inflammatory cytokines such as interferon-gamma. M2 macrophages are mainly involved in anti-inflammatory responses, ultimately associated with promoting wound healing, tissue repair and for resolving inflammation (31, 32). Glucocorticoids have been related to a M2-like phenotype, where the capacity to promote tissue repair and wound healing has been demonstrated (33–35). However, the direct participation of GR in the polarization still are not as well-understood.

Macrophages play a critical role in determining the extent of our body’s inflammatory response. However, macrophage function becomes impaired with increasing age and this could be linked to an imbalance between the amount of cortisol generated and the increase in the quantities of pro-inflammatory molecules produced in the body. Recently, has been proposed that low levels of the stress hormone cortisol and loss of the glucocorticoid-induced leucine zipper (GILZ) expression in macrophages can trigger chronic inflammatory responses in the body, contributing to the aging process (36).

Here, we review mechanisms whereby glucocorticoids can regulate physiological tissue homeostasis through the macrophage as a sensor, with emphasis on tissues where glucocorticoid signaling has been ablated using specific GR knockout mouse models. We propose that the pro-inflammatory or positive gene regulatory actions of glucocorticoids on macrophages may be a way in which macrophages shape the physiology of tissues.

## Glucocorticoids in the Immune-Surveillance of the Heart

Glucocorticoid signaling has direct effects during cardiac development and in both physiological and pathological conditions of the cardiovascular system. Multiple studies have revealed an important role for circulating glucocorticoids in the regulation of heart function and in impaired infarct healing, but they have not discriminated between direct and systemic actions of these hormones (37). By generating mice lacking GR expression solely in heart tissue (the cardiomyocyte-specific GR knockout or cardioGRKO), our group found that mice died prematurely from pathological cardiac hypertrophy that progressed to dilated cardiomyopathy and heart failure (38). It is established that endogenous glucocorticoids can also signal through the closely related mineralocorticoid receptor (encoded by Nr3c2, hereafter MR). For example, Oakley et al. generated mice lacking both GR and MR in cardiomyocytes which were resistant to cardiac disease in comparison to cardioGRKOs (39). Interestingly, these findings suggest that an appropriate amount of glucocorticoid signaling through both GR and MR in cardiomyocytes is critical for maintaining a healthy heart.

Heart failure is one of the leading causes of morbidity and mortality. It is recognized that innate immune cell activation occurs in patients with heart failure. This activation is associated with adverse clinical outcomes for disease progression. While it is accepted that neutrophils produce robust inflammatory responses and contribute to heart damage after acute ischemic injury, macrophages improve healing and cardiac remodeling after injury by promoting neutrophil efferocytosis, suppressing free radical formation, and modulating fibroblast activation state; however, the exact roles played by macrophages continue to be explored and defined (40–42). Paradoxically, macrophages can also trigger a damaging inflammatory response, which was shown in a zebrafish model where macrophages directly contributed to fibrosis during heart repair (43).

It has been suggested that distinct macrophage populations, such as resident or recruited subsets, may favor healing of injured areas or promote inflammatory and reparative functions (44). In the heart, tissue-resident macrophages populate different regions, including the ventricular myocardium, where they are found throughout myocardial interstitial spaces and interact directly with capillary endothelial cells and cardiomyocytes (45). They are also found in the atrioventricular node, where they facilitate electrical conduction by coupling to cardiomyocytes through connexin 43-containing gap junctions (46). Recently, Nicolas-Avila et al, demonstrated that macrophages can clean up dysfunctional mitochondria from cardiomyocytes, helping to maintain cardiac health and homeostasis (47). These data suggest broader homeostatic functions for heart resident macrophages; therefore, macrophages are an emerging target for therapeutic strategies aimed at minimizing cardiomyocyte death, ameliorating pathological cardiac remodeling, and treating heart failure after myocardial infarction.

Glucocorticoids play key roles in the regulation of macrophage homeostatic functions and in their functional properties to resolve inflammation and tissue damage (22). The loss of glucocorticoid-mediated regulation of macrophage function in the heart could result in the dysregulation of factors that control inflammation, neovascularization, collagen degradation, and scar tissue formation. In a model of myocardial infarction, mice lacking GR in myeloid cells under control of lysozyme M locus (LysM) promoter die earlier after infarction than wild type controls. GR-deficient macrophages were shown to exacerbate cardiac remodeling and to cause impairment of collagen scar formation and angiogenic response to ischemic injury, resulting in dysregulation of the resolution of inflammation and defects in wound healing (34).

**Figure 1:**
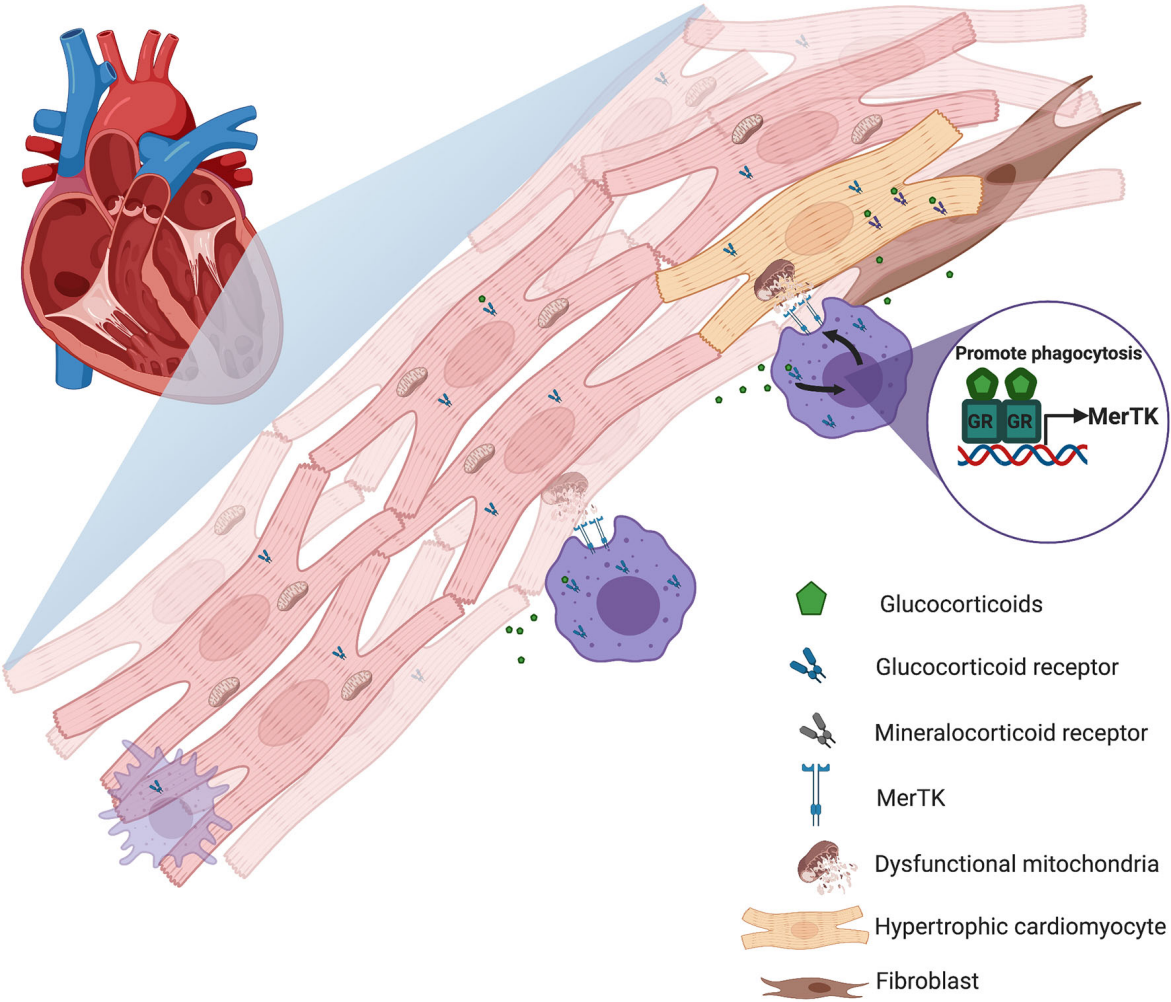
Schematic representation of how glucocorticoids could contribute to cardiac tissue homeostasis. Upon damage or stress activation, glucocorticoid-activated cardiac macrophages promote the active elimination of hypertrophic cardiomyocyte-derived mitochondria and help maintain cardiac health and homeostasis through the induction of the phagocytic receptor Mer tyrosine kinase (Mertk).

Finally, the newly discovered macrophage function related to the active elimination of cardiomyocyte-derived mitochondria through the phagocytic receptor Mer tyrosine kinase (Mertk) (47) reinforces the idea that glucocorticoids contribute to cardiac tissue homeostasis (**Figure 1**). It’s established that glucocorticoids upregulate Mertk expression in macrophages (48) and promote the phagocytosis of apoptotic neutrophils (49). The clearance of apoptotic cells and dysfunctional mitochondria by macrophages ensures mitochondrial and cardiomyocyte fitness, tissue proteostasis, and cardiac function. Therefore, glucocorticoids acting through macrophages could determine the balance between cardiac immunity and tolerance. Failure of this mechanism caused by defects in cardiac macrophage sensing of glucocorticoids, rather than from age related impairment of cardiomyocytes, could compromise cardiac homeostasis and promote heart disease.

## Glucocorticoids in the Immune-Surveillance of the Central Nervous System

The central nervous system (CNS) is a prominent target of glucocorticoids because GR is ubiquitously expressed in neurons, glial cells (such as astrocytes, oligodendrocytes and microglia) (50–53) in addition to brain vasculature (54). Moreover, differential expression of the glucocorticoid receptor has been described in specific subregions of the human cortex such as the basolateral amygdala, CA1 hippocampus and dentate gyrus. Glucocorticoid effects on the brain are related to the adaptation to stress. They primarily depend on GR distribution and functional pattern, and vary with gender, age, hormone concentrations, timing, and duration of exposure (55). There is compelling evidence for direct GC effects on behavior, cognition and mood (56, 57). At the cellular level, glucocorticoids are necessary for neuronal growth and differentiation. They also have an impact on several neuronal functions, including cell survival, integrity, and synaptic plasticity (58, 59). In humans and rodents, it has been described that glucocorticoids play a role in both embryonic and adult neurogenesis (60). Similar to embryonic development, neurogenesis in the adult happens in the hippocampus and involves a multi-step process starting with the division of neural stem cells and subsequent maturation into neural progenitor cells, proliferation of progenitor cells, maturation, morphological changes, migration, physiological adaptation, and functional integration into the hippocampal network (61). Newly generated neurons in the hippocampus contribute to learning and memory (62), forgetting (63) and cognitive flexibility (64). Interestingly, chronically elevated glucocorticoid levels under prolonged exposure to stress has been related to changes in the hippocampal cytoarchitecture, such as atrophy of dendritic processes and inhibition of neurogenesis (65, 66). Hippocampal neurons also play an essential role in the negative feedback regulation of the HPA-axis (67). Consequently, impaired hippocampal neurogenesis is closely associated with brain disorders and neurodegeneration by disrupted hypothalamic-pituitary axis functions. Interestingly, Quarta C., et al. (68), developed a tissue-specific anti-inflammatory drug that conjugate glucagon-like peptide-1 (GLP-1) to dexamethasone (GLP-1/Dexa) to selectively delivers dexamethasone to GLP-1 receptor (GLP-1R)-expressing cells (68). They showed that GLP-1/Dexa ameliorates the diet-induced systemic inflammation and does not induce negative effects on HPA-axis activity however, they do not deepen into the identity of the GLP-1R-expressing cells (68). Some studies have shown that macrophages could be one of the cells responding to the drug because they also express GLP-1R (69, 70). In addition, we recently published that dexamethasone induced the expression of the exopeptidase DPP4 that is recognized as one of the most important inactivator of GLP1 (21).

Glucocorticoids also play a crucial role in regulation of the immune system and intermediate metabolism within the brain. By inhibiting the immune system, glucocorticoids prevent overproduction of inflammatory molecules that can be harmful to neurons. The most notable CNS immune cells affected by glucocorticoids are the microglia. As resident macrophages of the nervous system, microglia are the brain’s professional phagocytes that sense and coordinate the brain inflammatory response. Microglia are the predominant immune cells of the CNS, comprising approximately 10–12% of the cells in the brain, with higher numbers within the hippocampus (71). Microglia normally exist in a quiescent or “resting” state in the healthy adult brain and, in response to tissue injury or disease, can transform rapidly from a quiescent state to different activation states (72). They are highly motile cells that survey the local environment and release cytokines that coordinate the response of both innate and adaptive immunity to control infection, remove cell debris and promote tissue repair (73). Upon activation, microglia upregulate cell surface molecules including major histocompatibility complex class I and II, receptors for cytokines and chemokines, such as CD200R (74) and CX3CR1 (75), and several other cellular markers indicative of increased reactivity (76). The constitutive expression of HLA-DR in human microglia has been related to their immune-surveillance of the brain (77). Recently, it has been described that microglial mTOR-dependent metabolic flexibility and glutaminolysis support their effector functions within the brain parenchyma (78).

In addition to their roles as immune sentinel cells, microglia also play a direct role in the regulation of neuron networks and physiology. Microglia can produce factors that modulate proliferation or survival of neurons (79, 80). Consistent with the well-known microglia functions as a sensors and phagocyte cells, Wang et al. (81), demonstrated that microglia eliminate synaptic components in the adult hippocampus, leading to dissociation of engram cells and the forgetting of previously learned contextual fear memory in a complement- and activity-dependent manner.

**Figure 2:**
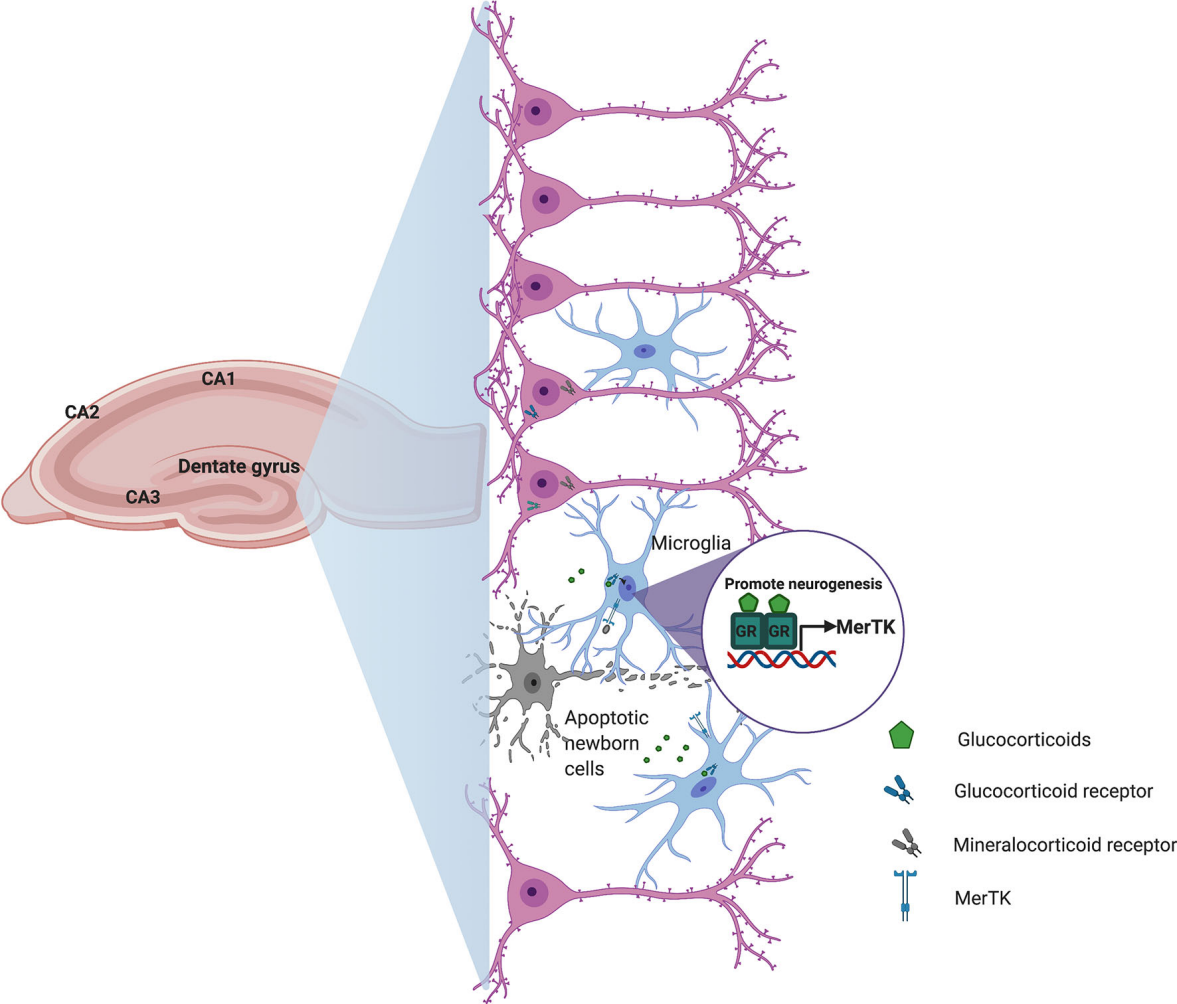
Schematic representation of how glucocorticoids could contribute to central nervous system homeostasis. During hippocampal neurogenesis, cell debris derived from apoptotic newborn cells and stress-induced glucocorticoid secretion promote microglia activation and transcriptional induction of the phagocytic receptor Mertk to regulate the production of new neurons in order to maintain homeostasis in the adult hippocampal neurogenic niche.

Conditions that are commonly associated with microglial activation and inflammation in the brain, such as aging, chronic stress, and neurodegenerative diseases also affect adult hippocampal neurogenesis (82). Mechanisms of immune regulation in the CNS are largely dependent on neuronal viability and activity, so the interactions between neurons and microglia are essential in maintaining brain homeostasis (83). Recently, Diaz-Aparicio et al. (84) showed that microglia also modulate adult hippocampal neurogenesis through the secretome associated with phagocytosis of apoptotic newborn cells *via* purinergic P2Y12 receptor and MerTK. As we discussed above for heart immunosurveillance, glucocorticoids could also regulate the expression of MerTK on microglia and promote the long-term homeostasis of adult hippocampal neurogenesis (**Figure 2**).

The CNS is highly sensitive to damage and any inflammatory response occurring within this organ system must be regulated. Microglial activation, and subsequent suppression, is necessary for host defense and neuroprotection following insult. However, these microglial neuromodulatory mechanisms may become deficient and/or dysregulated under excessive or prolonged inflammatory stimulation induced by stress, disease, and injury (71). One proposed mechanism for maintaining control of microglial activation is through their interaction with neuronal signaling molecules. Healthy neurons maintain microglia in their resting state *via* secreted and membrane bound signals, including CD200, CX3CL1 (fractalkine), neurotransmitters and neurotrophins (83, 85). A reduction in these regulatory factors can lead to microglia hyperactivation, suggesting an important role for communication between neurons and microglia in regulating neuroinflammation.

Although it has been known that glucocorticoid signaling is required for proliferation, differentiation, and survival of neurons, it appears to also be a critical regulator of microglia immunosuppression. By inhibiting microglial activation, glucocorticoids may have opposite effects in changing the immune status of the brain and may make neurons more susceptible to damage. In contrast, studies have suggested that GR activation in microglia promotes their neuroprotective function (86, 87). For example, GR-deficiency in microglia exacerbated neuronal and axon damage caused by intraparenchymal injection of LPS, and GR signaling in microglia suppresses stress-induced neuronal death (86). Interestingly, Maatouk et al. demonstrated that the number of microglia-expressing GR was significantly reduced in the brain of post-mortem Parkinson’s disease subjects compared to control tissue and also observed a significant upregulation of TLR9 protein (87). Moreover, in two mice model lacking GR in microglia/macrophages (GRLysM^cre^ and GRCX3CR1^CreER2^ GR mutant mice), intranigral injection of CpG-ODN (TLR9 ligand), resulted in significant loss of dopamine neurons in the brain (87). Although is well known that glucocorticoids are key regulators of TLRs activation upon inflammation, these data suggesting that the loss of GR in microglia also could contribute to dopamine neurodegenerative process. While both increased microglia activation and neuronal injury can be the result of an exaggerated neuroimmune response, it is unknown if microglial overactivation precedes and causes neuronal damage, or if activation occurs in response to loss of normal neuronal integrity. The differential and separate effects of glucocroticoids on neurons and microglia might depend on the machinery each type of cell possesses, the timing of exposure (before, during, or after activation) and the way they ultimately integrate permissive, preparative, suppressive, and stimulatory effects.

## Glucocorticoids in the Immune-Surveillance of the Gastrointestinal Tract

The gastrointestinal (GI) tract represents the largest interface between the organism and the external environment. The GI tract is persistently exposed to a high antigenic load derived from the dense, but largely harmless, commensal microbiota. Because of the mutualistic relationship between microbiome and host, the GI tract establishes a delicate coupling of immune resistance to pathogens and tolerance to tissue damage and inflammation. An important player in this process is the tissue macrophage. Intestinal macrophages, which function as phagocytes, are crucial to maintain the homeostasis of normal healthy GI tract tissues, but are also important for protection against pathogens through the secretion of pro-inflammatory mediators. Intestinal macrophages are also involved in the repair of damaged tissue through the production of proteins that drive epithelial cell renewal (88–90). Tissue-resident macrophages in the steady state are strongly influenced by the microbiota, and major populations are distributed in the stomach as well as along the length of the small and large intestines (91, 92). Unlike many other tissue macrophages, those in the mucosa of the GI tract are derived by continuous but distinct replenishment rates from circulating monocytes (27, 93). Interestingly, a new population of self-maintaining macrophages that are closely positioned in the intestinal submucosa and muscularis externa and arise from both embryonic precursors and adult bone marrow-derived monocytes, persists throughout adulthood and promotes intestinal homeostasis (94). At the functional level, these self-maintaining macrophages control intestinal physiology by supporting the vascular architecture, the permeability, and the intestinal motility that regulates neuronal function in the myenteric plexus (94).

**Figure 3:**
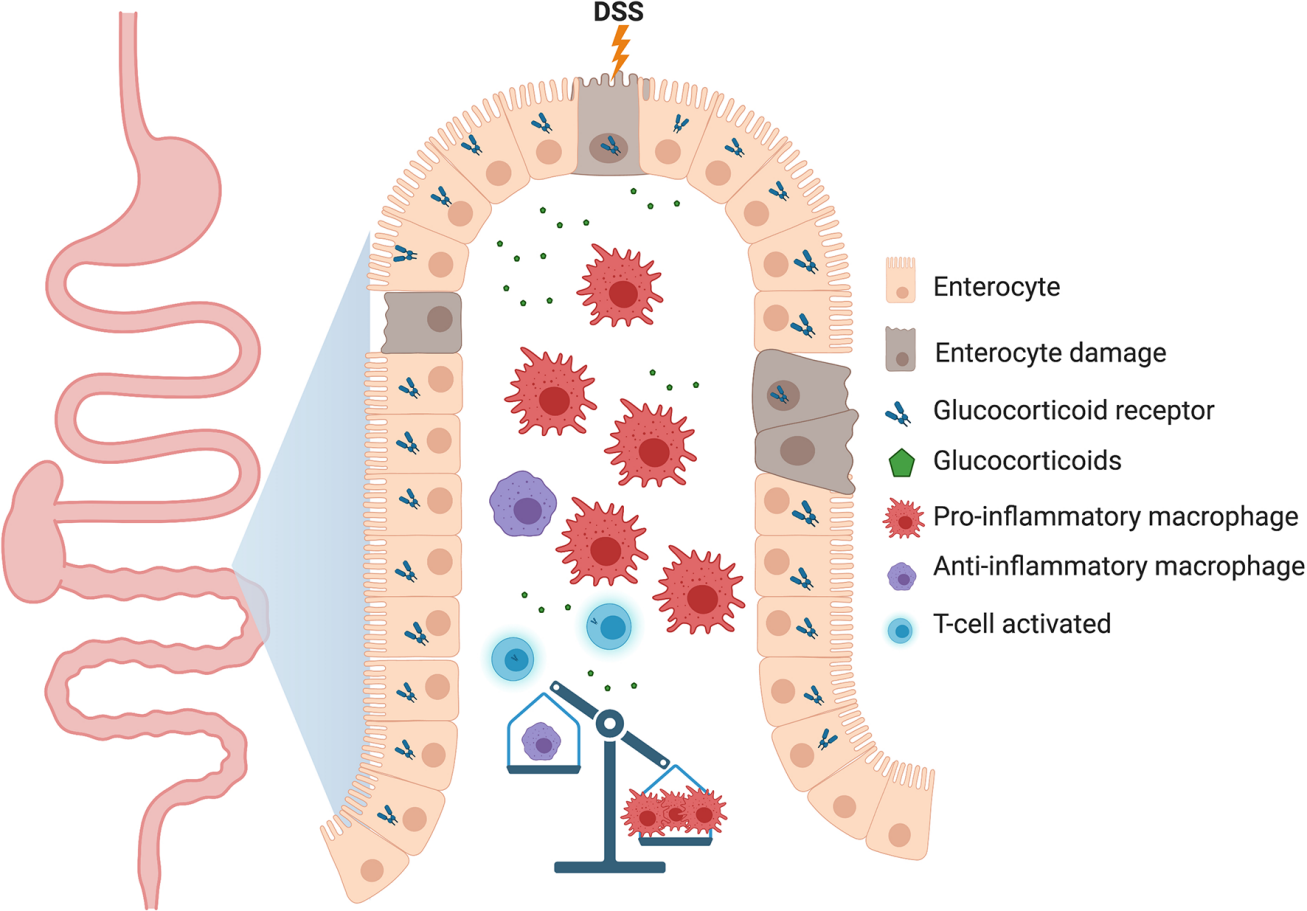
Proposed model of how the lack of glucocorticoid receptor (GR) in macrophages could be detrimental to gastrointestinal tissue homeostasis. In the dextran sodium sulfate (DSS)-induced colitis model, deletion of GR in myeloid cells delays the resolution of inflammation through an increase in the number of pro-inflammatory macrophages which perpetuates tissue damage. Other immune cells, such as B cells, dendritic cells and ILCs relevant to the intestinal physiology have been deliberately neglected to highlight the function of macrophages.

The important role of resident gastrointestinal macrophages in maintaining local homeostasis was discovered through a study by Zigmond et al. (95), where mice harboring IL-10 receptor alpha subunit (IL10RA) deficiency failed to sense interleukin-10 (IL-10; a pleiotropic and anti-inflammatory cytokine produced by T cells, B cells, and macrophages upon inflammation), resulting in spontaneous development of severe colitis. In a mouse model of inflammatory bowel disease (IBD), a chronic inflammatory disorder of the GI tract, mice lacking GR in myeloid cells (GRlysM) displayed impaired disease resolution to dextran sulfate sodium (DSS)-induced colitis and a diminished expression of IL-10 (35). The defect in the acquisition of an anti-inflammatory status and the lack of tissue repair caused by GR ablation in myeloid cells was characterized by persisting clinical symptoms and tissue damage, demonstrating an essential role for GR in macrophages for the induction of tissue repair mechanisms after intestinal tissue damage (**Figure 3**).

Glucocorticoid signaling in the GI tract plays a role in both regulation of the intestinal stress response and intestinal tissue homeostasis. This is suggested by the fact that synthetic glucocorticoid therapy is effective in inducing remission in IBD patients (96). Elevated GC levels as a result of stress or treatment also enhance the nutrient absorption by enterocytes (97, 98). Moreover, an increase in gastric acid secretion, induction of gastroparesis or gastric emptying, and the possible formation of gastric ulcers, in addition to enhanced intestinal glucose transport, have been observed after GC treatment (99). In a study using GRvillinCre mice, Reichardt et al. (100) demonstrated that the lack of GR in enterocytes did not protect mice from glucocorticoid-induced gastroparesis, suggesting that this pathology could be mediated directly by GR in the stomach. Moreover, Cipriani et al. demonstrated that proinflammatory macrophages were necessary for the development of gastroparesis in diabetic mice (101), suggesting that glucocorticoid signaling in the epithelium is needed to control macrophages activation.

Therefore, it is conceivable that GC effects on enterocytes might also contribute to the homeostasis of the entire GI tract. The role of glucocorticoid signaling in the stomach was recently investigated by our group through the depletion of circulating glucocorticoids in mice by adrenalectomy (ADX) (102). The lack of systemic endogenous glucocorticoids in mice resulted in the rapid onset of spontaneous gastric inflammation and the appearance of a clinical phenotype of spasmolytic polypeptide expressing metaplasia (SPEM), a precursor of gastric cancer (102). Moreover, the SPEM which developed in ADX mice was prevented by clodronate treatment and within the Cx3cr1 knockout mouse model, indicating that CX3CR1+ macrophages derived from monocytes are critical mediators of gastric inflammation (102). Intriguingly, the adrenalectomy does not trigger inflammation within another section of the stomach (gastric corpus greater curvature) and neither in other sections of the GI tract, such as ileum and colon (102). Interestingly, both small and large intestinal mucosa have been recognized as site of extra-adrenal glucocorticoid synthesis (103–105). One of the plausible reasons why ADX mice would not develop spontaneous inflammation in the intestine could be the local production of GCs. In order to cope the local stress, GCs would regulate the immune homeostasis, however this hypothesis have not been addressed yet. The nuclear receptor liver receptor homologue-1 (LRH-1, NR5A2) would be essential to regulate the intestinal glucocorticoid synthesis *in vivo* (104). Moreover, thought 3 different models, human intestinal organoids, humanized murine intestinal organoids, and a humanized murine IBD model Bayrer al. showed that LRH-1 promotes normal intestinal epithelial homeostasis suggesting that this NR can be an important regulator of intestinal tissue integrity (105). Summarizing, all these findings indicate that glucocorticoid signaling could participate in the immune-surveillance of the gastrointestinal tract and is a critical mediator of both gastric and intestinal homeostasis.

## Glucocorticoids in the Immune-Surveillance of the Liver

As their name suggests, glucocorticoids are profound regulators of glucose metabolism. It is no surprise then that the liver, the organ responsible for controlling glucose levels, is a major target of glucocorticoid action. Glucocorticoids exert permissive effects on glycogen metabolism and stimulate gluconeogenesis through direct regulation of rate limiting enzymes involved in this process, such as PEPCK and G6Pase (106, 107).

Studies using liver-specific GR knockout (L-GRKO) mice have demonstrated the many roles played by GR signaling in hepatocytes. The daily rhythmic production of endogenous glucocorticoids coordinate glucose, lipid, and fatty acid metabolism with periods of feeding and fasting (108, 109). This coordination is lost in L-GRKO mice, which exhibit fasting hypoglycemia and reduced body weight, which may involve impaired growth hormone signaling (110, 111). Our group evaluated the actions of glucocorticoids on hepatic expression of inflammatory genes in male and female L-GRKO mice. Interestingly, in response to pro-inflammatory LPS challenge, deletion of GR reduced the number of inflammatory genes in a sex-specific manner, with female mice exhibiting regulation of more genes than males (112). Therefore, GR signaling influences diverse gene expression programs in hepatocytes, some of which are sexually dimorphic.

In addition to its roles in regulating metabolism, the liver is also an important part of the immune system. It serves as a major filter for the blood coming from the digestive tract and can respond to potential threats such as bacterial toxins and cellular debris.

Hepatocytes release large amounts of immunoregulatory proteins into the circulation that function to eliminate pathogens and fine-tune innate immunity. These include members of the complement system, acute-phase proteins, LPS signaling regulators, and several iron-metabolism-related proteins (reviewed in (113)). The liver also contains the single largest reticuloendothelial cell network in the body, which is composed of tissue-resident macrophages called Kupffer cells. Kupffer cells are intimately involved in the hepatic response to various toxic insults. They constitute a primary line of defense against invading microorganisms, function as sensors for altered tissue integrity, and control immunological tolerance in the liver by providing an anti-inflammatory microenvironment during homeostasis (114). Kupffer cells are largely stationary and adhere to the liver sinusoidal endothelial cells where they are exposed to the contents of the blood. During times of inflammation, the hepatic macrophage pool is expanded by circulating blood monocytes that give rise to monocyte-derived macrophages (115), which have been shown to resemble the transcriptional phenotype of Kupffer cells after lymphocyte choriomeningitis virus infection (116) or after acetaminophen-induced hepatotoxicity (117).

**Figure 4:**
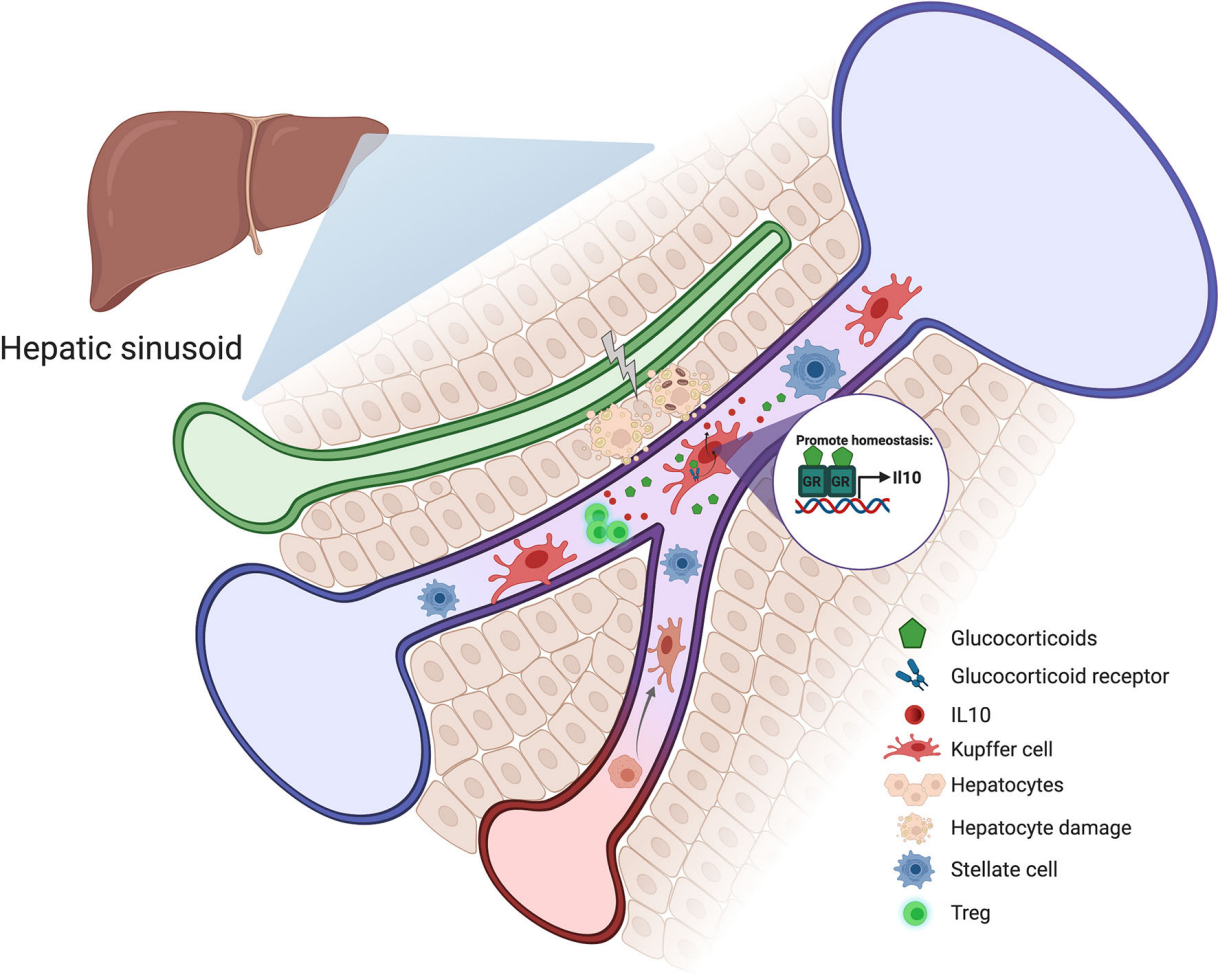
Schematic representation of how glucocorticoids could contribute to liver homeostasis. Stress and hepatocyte injury around the hepatic sinusoid cause an increase in glucocorticoid levels which induces Kupffer cell activation and IL-10 production. Kupffer cell-derived IL-10 exerts hepatoprotective and tolerogenic effects through Treg activation.

Relatively few studies have explored the role of GR signaling in Kupffer cells and monocyte-derived liver macrophages. Nevertheless, there is good evidence that glucocorticoid regulation of these immune cells may be essential for liver homeostasis. Kupffer cells respond to glucocorticoids by upregulating the anti-inflammatory gene Gilz, and mice with macrophage-specific deficiency in GR exhibit more severe obesity-induced liver inflammation (118). In human and mouse, Kupffer cells secrete the immunosuppressive cytokine IL-10 (119). IL-10 deficiency or depletion exacerbates hepatic immune-mediated liver damage and abrogates tolerance induction (120). For example, in the Concanavalin A hepatitis model, which is used to study tolerance induction and immune-mediated hepatitis, Kupffer cell-derived IL-10 exerts hepatoprotective and tolerogenic effects through Treg activation (121). Similarly, Kupffer cells interact directly with T cells in response to administration of particulate antigens, causing the expansion of IL-10-expressing Tregs (122). It is well known that IL-10 is a glucocorticoid-induced gene (123, 124); therefore, glucocorticoid levels may directly influence the inflammatory environment in the liver by modulating Kupffer cell IL-10 expression (**Figure 4**).

The liver’s response to injury is complex and depends upon the interaction of multiple cell types, both parenchymal and non-parenchymal. Paradoxically, Kupffer cells have been implicated in both liver regeneration and fibrosis. They secrete pro-inflammatory mediators, such as reactive oxygen species, eicosanoids, prostaglandins, and cytokines that induce recruitment of additional inflammatory cells to the liver (125).

Glucocorticoid receptor ligands have been shown to suppress hepatic expression of pro-fibrotic genes, leading to decreased extracellular matrix deposition (126). In addition, GR ligands inhibit immune cell infiltration to the damaged liver, which can exacerbate injury in certain cases. Using models of tissue-specific deletion of GR, Kim et al. (126) demonstrated that GR signaling controls pro-fibrotic gene expression and immune cell infiltration *via* two cell types. Specifically, GR deletion in Kupffer cells (via LysM-cre) reversed inhibition of immune cell infiltration in response to dexamethasone. GR deletion in hepatic stellate cells (via hGFAP-cre) reversed downregulation of fibrotic gene expression in response to dexamethasone. These studies suggest that GR signaling in Kupffer cells modulates factors involved in cell recruitment to the liver, while GR signaling in hepatic stellate cells modulates fibrosis in response to injury. Other studies have also supported the idea that GR signaling in Kupffer cells promotes liver homeostasis. Direct targeting of dexamethasone to Kupffer cells promoted replenishment of glycogen stores lost during hepatic fibrosis caused by bile duct ligation (127). Interestingly, Rose et al. (128) demonstrated that the use of glucocorticoids completely sustained hepatocyte longevity and improved hepatocyte functionality during the establishment of co-culture conditions between hepatocytes and Kupffer cells. Additional studies are needed to pinpoint the effects of both endogenous and synthetic glucocorticoids on specific cells within the liver and to understand the interaction between these cell types during times of injury and homeostasis.

## Conclusions and Perspectives

Glucocorticoids mediate physiological processes in different tissues and cell types with high specificity to systematically influence behavior and cognition, metabolism, cardiovascular function, and the immune system. Synthetic glucocorticoids are administrated as drugs to treat several inflammatory conditions because of their ability to induce potent anti-inflammatory and immunosuppressive effects that occur due to the repression of pro-inflammatory genes and the activation of anti-inflammatory pathways in immune cells. Whereas restricted inflammation is beneficial, excessive or persistent inflammation could be associated with chronic diseases. The immune regulation process and anti-inflammatory homeostatic mechanisms mediated by glucocorticoids are essential in limiting and resolving the inflammatory process. The balance of pro- and anti-inflammatory pathways plays an important role in maintaining immune homeostasis. In addition to its immunosuppressive functions, GR signaling may regulate cellular metabolism and survival.

The main mechanism of action of GCs on immune cells has been linked to their ability to induce cell death and reduce cell survival through direct genomic effects. GR-regulated genes that are required to drive apoptosis include pro-apoptotic mediators, such as the BH3-protein BIM (BCL2LII), which is activated (129) and the anti-apoptotic BCL2, which is downregulated (130). Non-genomic effects of GR have also been proposed. Interestingly, a second mechanism that could explain the effectiveness of GC regulation of the immune response is the promotion of Treg proliferation directly or indirectly through macrophage activation. Bereshchenko et al. (131) reported that glucocorticoid-induced leucine zipper (GILZ) promotes Treg production and enhance Treg signaling. Macrophages have been shown to possess the potential to induce Treg function to maintain tissue homeostasis, while Tregs can enhance the ability of macrophages to engulf apoptotic cells which promotes resolution of inflammation (132). Disruption of the crosstalk between macrophages and Tregs leads to severe autoimmune disease and chronic inflammation.

The glucocorticoid regulation of local homeostatic mechanisms has been exemplified through the study of tissue-specific GR knockout mice. We still have limited knowledge of how GR-dependent gene expression contributes to the phenotypes of tissue-specific GR knockout mice. Future studies are needed to fully understand how GR signaling is acting in specific tissues and in different disease states.

Its well-known that glucocorticoids can regulate different stages of macrophage biology, including differentiation, survival, movement, activation and polarization. While it has been long believed that tissue macrophages were originated from myeloid cells and circulating adult blood monocytes, it is now clear that many resident tissue macrophages are established during embryonic development and persist by self-renewal. Even, we now know that multiple populations of macrophage-like cells co-exist in both, steady-state and inflammation. Moreover, under inflammatory conditions, the macrophage pool is expanded by pro-inflammatory infiltrating blood monocytes that may or may not acquire the phenotype of the resident macrophages in a given tissue. Whether glucocorticoids can regulate the gene expression profile of macrophages independently of their ontogeny, activation or polarization states is one of the most important questions that must be addressed. Uncovering distinct glucocorticoid-mediated gene expression networks in macrophages may aid in the production of targeted therapies for diseases characterized by dysregulation of homeostasis.

## Author Contributions

DD-J and JK wrote the manuscript with supervision from JC. All authors contributed to the article and approved the submitted version.

## Funding

This work was supported by the Intramural Research Program of the NIEHS, National Institutes of Health (1ZIAES090057). This work was also supported by postdoctoral fellowship from NIH (Award #76146) (to DD-J). The content is solely the responsibility of the authors and does not necessarily represent the official views of the National Institutes of Health.

## Conflict of Interest

The authors declare that the research was conducted in the absence of any commercial or financial relationships that could be construed as a potential conflict of interest.
